# Task-Specific Balance Training Improves the Sensory Organisation of Balance Control in Children with Developmental Coordination Disorder: A Randomised Controlled Trial

**DOI:** 10.1038/srep20945

**Published:** 2016-02-11

**Authors:** Shirley S.M. Fong, X. Guo, Karen P.Y. Liu, W.Y. Ki, Lobo H.T. Louie, Raymond C.K. Chung, Duncan J. Macfarlane

**Affiliations:** 1 Institute of Human Performance, The University of Hong Kong, Pokfulam, Hong Kong; 2 Department of Rehabilitation Sciences, The Hong Kong Polytechnic University, Hung Hom, Hong Kong; 3 School of Science and Health (Occupational Therapy), University of Western Sydney, NSW, Australia; 4 Health, Physical Education and Recreation Department, Emporia State University, USA; 5 Department of Physical Education, Hong Kong Baptist University, Kowloon Tong, Hong Kong

## Abstract

Sensory organisation of balance control is compromised in children with developmental coordination disorder (DCD). A randomised controlled trial involving 88 children with DCD was conducted to evaluate the efficacy of a task-specific balance training (functional-movement training, FMT) programme in improving balance deficits in a DCD population. The DCD participants were randomly assigned to either a FMT group or a control group. The FMT group received two training sessions/ week for 3 months. Measurements of the participants’ sensory organisation (somatosensory, vestibular and visual ratios), balance and motor proficiency (Movement Assessment Battery for Children, MABC scores) and center of pressure sway velocity (Unilateral Stance Test, UST scores) were taken at baseline, immediately after FMT and 3 months after FMT. The FMT group showed greater improvements than the controls in somatosensory ratio at 3 and 6 months (all P < 0.001), but the within-group changes were not significant (P > 0.05). The results of both the MABC and the UST also indicated that the balance performance of the FMT group was significantly better than that of the control group at 3 and 6 months (all P < 0.05). Task-specific balance training was found to marginally improve the somatosensory function and somewhat improve the balance performance of children with DCD.

Developmental coordination disorder (DCD) is one of the most common childhood neurodevelopmental movement disorders, affecting about 6% of typically developing children. The disorder is characterised by the significant impairment of motor skills, including balance skills^1^. Indeed, balance dysfunction is one of the most common sensorimotor disorders exhibited by this group of children, with a prevalence rate of between 73% and 87%^2^. It is important to study balance function because suboptimal balance may increase children’s risk of falls, hamper their motor-skills development^3^ and limit their participation in activities^4^,^5^.

To maintain body balance, the inputs supplied by three sensory systems (somatosensory, visual and vestibular) must be organised and the correct sensory signals selected to generate coordinated movements^6^. Children with DCD exhibit deficits in sensory organisation, especially in utilising visual and vestibular inputs^7^,^8^ and re-weighting (increasing the use of) somatosensory inputs to ensure body balance^3^,^9^. Sensory re-weighting refers to the process of integration of sensory information utilized for postural control which is dynamically regulated to adapt to changing environmental conditions and availability of the three sensory signals^10^. Re-weighting of sensory information is particularly important under changing environmental conditions (e.g., walk in the dark) or when there is a loss of sensory functionality (e.g., blindness)^10^. Therefore, children with DCD demonstrate inferior balance performance when standing in sensory challenging environments^4^,^7^. The results of our previous study suggested that Taekwondo training can improve the use of visual and vestibular inputs to maintain body balance^11^, but no effective strategy has yet been identified to improve the sensory re-weighting ability of children with DCD. Sensory re-weighting plays a particularly important role in maintaining postural stability and safety in daily environments that present sensory challenges and so is very important for children with DCD^3^,^6^.

‘Task-oriented’ treatment is currently the most common method used to improve the motor skills and thus the balance performance of children with DCD^12^. This treatment strategy is based on the principles of motor learning and neuroplasticity^13^. Building on the task-oriented approach, researchers have developed an even more promising treatment strategy: ‘task-specific’ intervention^14^. The key principle of this treatment is to expose the child repeatedly to a given (balance) task under the right constraints (e.g., the child’s natural environment)^15^. A number of studies have shown that task-specific intervention can improve the motor performance of children with DCD in hopping, skipping and various balance activities^16^,^17^. However, no study to date has investigated the effectiveness of task-specific intervention in improving the sensory organisation of balance control, including sensory re-weighting ability, in the DCD population. The aim of this study was to assess the efficacy of a novel task-specific balance training programme – namely a functional movement training (FMT) programme – in improving the sensory organisation and balance control of children with DCD. We hypothesised that the members of the FMT group would exhibit greater improvements in sensory organisation and balance control during functional tasks than the control-group participants, whose members received no training.

## Results

### Study population

**Figure 1:**
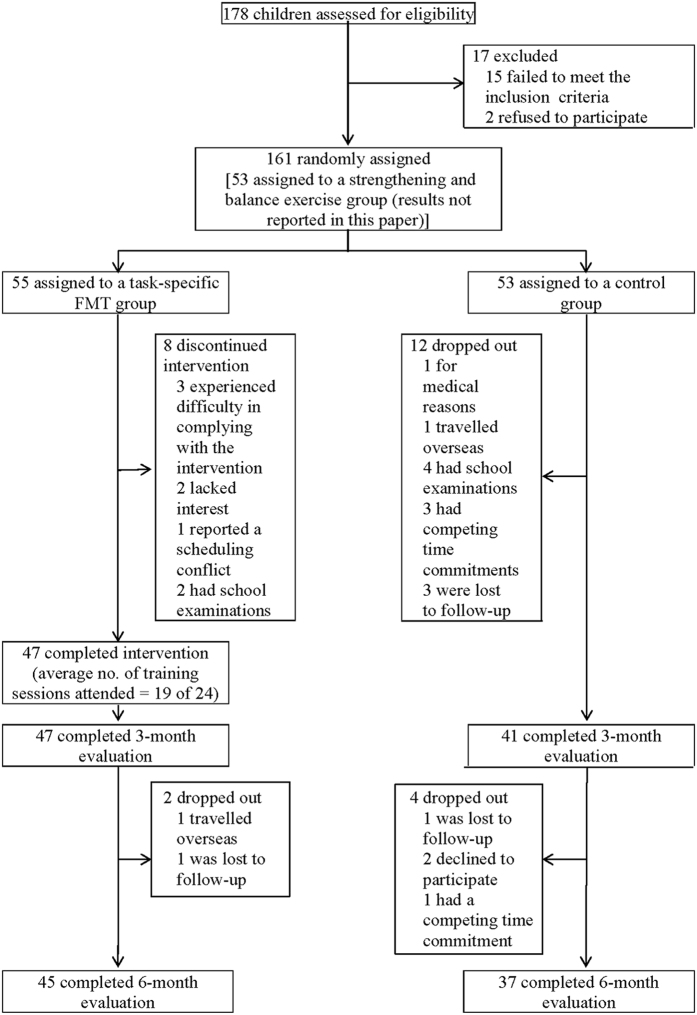
Participant flow.

Between January and May 2014, 178 children were screened for eligibility. Of the 161 eligible children with DCD, 55 were randomly assigned to a task-specific FMT group, 53 to a no-training control group and 53 to a strengthening and balance exercise group (results not reported in this paper). The children in both the FMT group and the control group had participated in our previous study. The flow of the participants through the stages of the randomised study is shown in Fig. 1. Table 1 presents a full set of baseline demographic data for the two groups of participants. No significant differences between the two groups were observed. In addition, there were no significant differences in baseline demographic variables between the participants who completed the trial and those who did not. The average participation rate in the FMT intervention was 79%. All of the participants attended 19 sessions (80%) or more. There were no within-group changes in the participants’ physical-activity levels or medication used during the study, and none of the participants received non-study intervention treatment.

### Primary outcomes

The results (equilibrium scores) of the Sensory Organization Test (SOT) can be found as Supplementary Table S1 online. SOT sensory ratio analyses indicated that, compared with the control group, the FMT group showed a greater improvement in somatosensory ratio at 3 months (0.03 points; 95% CI, 0.02 to 0.04; P < 0.001) and 6 months (0.03 points; 95% CI, 0.01 to 0.05; P < 0.001). However, the within-group changes were not significant. The SOT vestibular and visual ratios remained stable over time in both groups (Table 2). A separate analysis (on-protocol analysis) was performed after removing the data collected from the participants who dropped out of the study, and similar results were obtained (data not shown).

### Secondary outcomes

At 3 months, the FMT group achieved better results for functional balance than the control group in both the Movement Assessment Battery for Children (MABC) (between-group difference in balance subscores: −0.93 points; 95% CI, −1.42 to −0.44; P < 0.001) and the Unilateral Stance Test (UST) (between-group difference in centre of pressure sway velocity: −0.54 points; 95% CI, −0.91 to −0.16; P = 0.006). The improvement shown by the FMT group relative to the control group was maintained for 6 months, with a between-group difference of −0.83 points in the MABC balance subscores (95% CI, −1.52 to −0.14; P = 0.019) and −0.56°/s in UST centre of pressure sway velocity (95% CI, −0.92 to −0.20; P = 0.003). However, no significant within-group changes were observed in either group. In addition, no within-group or between-group changes were detected in the MABC total impairment score (TIS) during the 6-month study (Table 2).

### Adverse events

No major adverse events were reported during the intervention or the laboratory assessments. The adverse events reported during training, such as transient muscle soreness (*n* = 2) and non-injurious falls (*n* = 1), were minor.

## Discussion

This study yielded the novel finding that a 3-month programme of twice-weekly task-specific balance training (in the form of an FMT programme) improves the sensory organisation of balance control in children with DCD by increasing their reliance on somatosensory information for balance. A concomitant improvement in functional-balance performance was indicated by a decrease in both MABC balance subscores and UST centre of pressure sway velocity after training. All of these improvements were maintained for 3 months after the intervention period. No serious adverse events were observed, indicating the safety and usefulness of this intervention for the target population. Our findings are actually in line with a previous study showing that a 9-week task-specific intervention (target kicking) resulted in significant improvement in performance of the target kicking task in clumsy children^16^. The present findings further suggest that it might be related to an improvement in sensory organization ability.

Our findings are indeed encouraging, as they suggest that the proposed task-specific intervention is a safe and effective treatment for sensory-organisation and balance disorders in children with DCD. DCD is widely acknowledged to impair children’s ability to utilise visual and vestibular inputs for body balance^3^,^7^,^8^ In addition, children with DCD find it difficult to maintain their body balance by re-weighting somatosensory information (to compensate for visual and vestibular deficits)^3^,^9^,^18^. Therefore, none of the three sensory inputs provide accurate and reliable tools for postural control, inevitably compromising functional-balance performance^6^.

The results of this study indicate a viable solution to this ongoing problem faced by the DCD population. The proposed task-specific balance training programme may enhance DCD-affected children’s ability to re-weight their relatively normal somatosensory input (DCD: SOT somatosensory ratio = 0.95 – 0.96 vs. normal: SOT somatosensory ratio = 0.96 – 0.97^11^) for balance control. This might improve aspects of their functional-balance performance, such as their stability while standing on one leg^6^. Having said that, our results may be interpreted in a different way – we found that the FMT group improved more than the control group in terms of somatosensory function, but still, there was no significant within-group improvement. It is plausible that the control group deteriorated in somatosensory function and FMT prevented the deterioration of somatosensory function and the associated balance performance. Further studies are necessary to confirm the clinical significance of the possible improvement or maintenance of somatosensory function after FMT in children with DCD. Moreover, we observed no improvement in the participants’ ability to use vestibular and visual information to maintain postural stability after the task-specific balance intervention. As suggested in our previous study, Taekwondo training may offer a supplementary method of treating the vestibular and visual deficits of children with DCD^11^.

Although most of our results appear to be promising, we have thus far been unable to identify the underlying neurophysiological mechanisms by which task-specific balance training along with electromyographic biofeedback increased the participants’ reliance on somatosensory inputs to maintain their balance. We postulated that repeatedly practising task-specific balance manoeuvres and involvement of cognitive processing (adjunct biofeedback training) might induce neural plastic changes and cortical reorganisation in the developing cerebral cortex^19^. Indeed, a previous electrophysiological study has shown that task-relevant somatosensory information can encourage selective facilitation within the primary somatosensory cortex^20^. The somatosensory cortex may undergo neural plastic changes during/after task-specific balance exercises. Nevertheless, further neurophysiological and neuroimaging studies are necessary to explicate the precise role of the proposed task-specific balance intervention in improving the balance function of children with DCD by changing their brain activity and neuroplasticity.

We found improvements in functional-balance performances in children with DCD after FMT (MABC balance subscore = 1.87 and UST centre of pressure sway velocity = 2.00°/s). These improvements were maintained for 3 months after the intervention period (MABC balance subscore = 1.97 and UST centre of pressure sway velocity = 1.95°/s). Although the MABC balance subscore achieved the normal level (<5.00)^21^ in the FMT group after training, the UST centre of pressure sway velocity was still higher than typically-developing children of similar ages (1.71°/s)^11^. Further studies might modify the current FMT protocol to include more single-leg standing balance exercises and re-evaluate its effectiveness in improving postural control in children with DCD.

The study reported here has some limitations. First, the participants were not blind to the group assignment, due to the nature of exercise training. The optimism of highly motivated participants about the benefits of the training may have introduced bias to the results^22^. Second, the generalisability of task-specific training has been questioned^23^. It is unclear whether the improvements observed in balance and sensory-organisation ability would be replicated in other, non-laboratory environments (e.g., outdoor and clinical settings). Further studies should investigate whether these improvements are clinically meaningful/important and taking individual differences into account. Third, the SOT somatosensory ratio theoretically measures the reliance on both somatosensory and vestibular inputs for balance control because the vestibular sense cannot be eliminated in all SOT conditions^6^. Therefore, participants in the FMT group might have increased the use of both somatosensory and vestibular inputs to maintain standing balance compared to the control group at 3 and 6 months. Finally, we collected data from the participants for only 6 months, so the long-term effectiveness of this task-specific balance training programme for children with DCD has yet to be determined.

In conclusion, the proposed task-specific balance training was found to marginally improve the somatosensory function and somewhat improve the functional balance performance of children with DCD. However, it did not improve vestibular and visual contributions to postural control in this particular group of children.

## Methods

### Study design

This assessor-blinded, stratified, randomised, controlled clinical trial was registered at ClinicalTrials.gov (NCT02393404) in March 2015. The study protocol was approved by the Human Research Ethics Committee of the University of Hong Kong. Written informed consent was obtained from each participant and parent before the screening and data collection. All experimental procedures were carried out in accordance with the approved guidelines and Declaration of Helsinki for human experiments.

### Participants

Posters and online advertising were used to recruit children with DCD from hospitals, child-assessment centres, primary schools, non-government organisations and parents’ groups. The requirements for inclusion were as follows: a diagnosis of DCD consistent with the criteria provided in the Diagnostic and Statistical Manual of Mental Disorders IV^1^; a gross motor composite score lower than or equal to 42 in the Bruininks-Oseretsky Test of Motor Proficiency^24^ or a MABC TIS below the 5^th^ percentile^21^; a total score of less than 46 (5 – 7 years, 11 months old), less than 55 (8 – 9 years, 11 months old) or less than 57 (10 – 15 years old) on the 2007 version of the DCD questionnaire^25^; age between 6 and 10 years old; and attendance at a mainstream school. The exclusion criteria were as follows: a diagnosis of emotional, neurological or other movement disorders (comorbid attention deficit hyperactivity disorder, attention deficit disorder, dyslexia and suspected autism spectrum disorder were acceptable); significant congenital, musculoskeletal, cardiopulmonary or sensorimotor disorders capable of affecting balance performance; receipt of active treatment, such as alternative medicine; disruptive behaviour; or an inability to follow instructions accurately.

### Screening and randomisation

Two physiotherapists screened the volunteers during telephone conversations. Those deemed eligible were evaluated in person, and received a baseline assessment. The eligible participants with DCD were stratified by sex and randomly assigned to either a task-specific FMT group or a control group (Fig. 1). The randomisation was carried out by an independent researcher who was not involved in the subject-recruitment process. A random-number table was used to generate the allocation sequence and sealed opaque envelopes were used to ensure concealed allocation. Since group assignment was random, the baseline characteristics including MABC TIS and balance subscore were similar between the two groups.

### Intervention

The members of the task-specific FMT group received specific balance training accompanied by electromyographic biofeedback (an extrinsic form of feedback) to remediate their motor-learning difficulties^23^ and enhance their neuroplasticity and balance performance^13^,^26^. The task-specific FMT protocol, adapted from the balance-assessment items of the MABC (items 2 – 5)^21^, is presented in detail in Table 3. A specific electromyographic-assisted balance exercise (item 1) was included in the protocol to increase movement awareness and control cognitively, maximise motor learning and enhance central nervous system plasticity^26^. During training, a NeuroTrac MyoPlus 4 machine (Verity Medical Ltd., Hampshire, UK) was used to apply electromyographic biofeedback to the participant’s dominant leg (i.e., the leg used to kick a ball) while standing on a stability trainer (The Hygienic Corporation, Ohio, USA)^27^. The activity of the rectus femoris and gluteus maximus muscles was monitored by visual feedback signals (in the form of bar graphs with higher bars representing higher muscle activities, to provide the participants with visual information/ feedback on their performance)^28^, because these muscles are essential to hip balancing strategy^29^ and also affect ankle movements^8^. The participants learned to maintain their balance through coordinated hip- and ankle-joint movements. They were instructed to contract the agonistic hip muscle as fast as possible (above a pre-set threshold) when their balance was being disturbed in the anterior-posterior direction and then to relax the same muscle to avoid overbalancing. In addition, the participants received verbal feedback on their performance (knowledge of the results) at the end of every training session to accelerate the motor-learning process^30^.

All of the training sessions were supervised by a physiotherapist and conducted by a trained research assistant with a sports-coaching qualification. The children in the intervention group attended two face-to-face training sessions per week (1.5 hours/session) at the University of Hong Kong Health and Physical Activity Laboratory for 12 consecutive weeks^31^. The control group received no physical training during the study period because many types of exercise, other than FMT, might also improve motor proficiency in children with DCD^11^,^12^,^14^,^15^.

### Test procedures

The data collection was carried out by a physiotherapist and an assistant, both of whom were blind to the group allocation, at the Balance and Neural Control Laboratory of the Hong Kong Polytechnic University. All of the participants were assessed before the intervention (baseline), immediately after the intervention (3 months) and 3 months after the intervention (6 months).

### Demographics

The age, sex, body weight, height, comorbid conditions, medication, treatment received and exercise habits of each participant were recorded. Body-mass index was calculated for each participant by dividing body weight by the square of height. The participants’ physical-activity levels in metabolic equivalent hours per week were also estimated on the basis of exercise intensity, duration, frequency and the metabolic equivalent value assigned to each activity in the Compendium of Energy Expenditures for Youth^32^.

### Primary outcomes

The sensory organisation of balance control (the primary outcome) was assessed using the SOT, because this test has been shown to be valid and reliable for use with children^33^,^34^. Each participant was instructed to stand on the force platform of a computerised dynamic posturography machine (Smart Equitest, NeuroCom International Inc., Clackamas OR, USA), wearing a security harness to prevent falls. Foot placement (the base of support) was standardised according to the participants’ height. Next, each participant was exposed to the following six sensory conditions in sequence: condition 1 – accurate somatosensory, visual and vestibular inputs; condition 2 – accurate somatosensory and vestibular inputs only, with no visual input; condition 3 – accurate somatosensory and vestibular inputs and inaccurate visual input; condition 4 – accurate visual and vestibular inputs and inaccurate somatosensory input; condition 5 – accurate vestibular input, no visual input and inaccurate somatosensory input; and condition 6 – accurate vestibular input and inaccurate visual and somatosensory inputs. Three trials were conducted for each sensory condition (18 trials per participant). The computerised dynamic posturography machine captured each participant’s centre of pressure trajectory over the 18 trials and automatically generated an equilibrium score (ES) for each trial. The three ESs for each sensory condition were averaged, and the results were used to calculate the participant’s somatosensory ratio (mean ES of condition 2/mean ES of condition 1), visual ratio (mean ES of condition 4/mean ES of condition 1) and vestibular ratio (mean ES of condition 5/mean ES of condition 1). These three sensory ratios reflect the contribution of each sensory system to balance control. A sensory ratio close to 1 for a particular sense indicates that an individual relies predominantly on that sense for balance^6^,^35^. All three sensory ratios were used in the analysis.

### Secondary outcomes

The MABC was used to assess the participants’ functional-balance performance and motor proficiency (secondary outcomes). The MABC is widely acknowledged to be a standardised, validated and reliable instrument for measuring aspects of children’s motor performance, such as their balance^21^,^36^ The instrument consists of eight gross and fine motor tasks for each of four age bands (4 – 6 years, 7 – 8 years, 9 – 10 years and 11 – 12 years). The eight tasks are divided into three domains: manual dexterity, ball skills and static and dynamic balance. Among the balance tests are balancing on one leg, jumping, walking on tiptoe and tandem walking. The assessment procedures are described in detail by Henderson and Sugden^21^. The participants were assessed using the tests appropriate to their respective age-bands. The raw scores for the test items were summed to obtain a TIS, and the raw scores for the three balance items were summed to obtain a balance subscore. A lower score represented better motor (balance) performance^21^. The scores obtained for both the test items and the balance items were used in the analysis.

The abovementioned computerised dynamic posturography machine was used to administer the UST to measure the participants’ single-leg standing balance (another secondary outcome). A previous study has shown that the UST has a good test-retest reliability when administered to young people, with an intraclass-correlation coefficient of 0.77^33^. During the test, each participant stood on his/her dominant leg for 10 seconds. A standardised testing posture was adopted (arms by the side of the trunk and the hip of the free leg flexed at 45°). The computerised dynamic posturography machine recorded the participants’ centre of pressure sway velocity during single-leg standing. Three trials were performed for each participant, at 10-second intervals^35^. The mean centre of pressure sway velocity across the three trials was calculated for each participant and used in the analysis. A lower score indicated better single-leg standing balance performance.

### Statistical analysis

Based on the data collected during our previous study^11^ and pilot trial, an average effect size of 0.67 was used for the primary outcome measures. As an attrition rate of 25% was anticipated, with 80% power and a two-tailed significance level of 5%, a minimum of 45 participants per group were needed.

All of the statistical analysis was conducted using the Statistical Package for the Social Sciences version 20.0 (IBM, Armonk, NY). Descriptive statistics (mean ± standard deviation) were produced for all of the variables. A Kolmogorov-Simirnov test and a histogram were used to check the normality of the data. To handle the missing data, the intention to treat (last observation carried forward) assumption was made. Participants who completed the 3-month evaluation but did not complete the 6-month evaluation were also included in the analysis. The between-group differences in the baseline demographic and outcome variables were assessed using independent t-tests (for the continuous data) and the chi-square test (for the categorical data). Any changes in the primary and secondary outcome measures were quantified by subtracting the baseline scores from the post-intervention scores. The differences in each outcome measure from the baseline were analysed using two-way repeated measures analysis of variance (between-subject factor: group; within-subject factor: time) followed by post-hoc Bonferroni tests, as appropriate, with an overall significance level of 5% (two-tailed test).

## Additional Information

**How to cite this article**: Fong, S. S.M. *et al.* Task-Specific Balance Training Improves the Sensory Organisation of Balance Control in Children with Developmental Coordination Disorder: A Randomised Controlled Trial. *Sci. Rep.* **6**, 20945; doi: 10.1038/srep20945 (2016).

## Supplementary Material

Supplementary Information

## Figures and Tables

**Table 1:** Baseline characteristics (mean ± SD) of the participants with developmental coordination disorder.

Characteristics	Task-Specific FMT (n = 47)	Control (n = 41)	P value
Age (years)	7.9 ± 1.4	7.5 ± 1.6	0.171
Sex (number and %)			0.846
Male	33 (70.2%)	28 (68.3%)	
Female	14 (29.8%)	13 (31.7%)	
Weight (kg)	25.8 ± 8.1	24.5 ± 8.3	0.470
Height (cm)	125.2 ± 11.0	121.5 ± 11.2	0.124
Body-mass index (kg/m^2^)	16.1 ± 2.5	16.2 ± 2.7	0.856
Physical-activity level (metabolic equivalent hours/week)	15.6 ± 13.4	17.2 ± 13.9	0.579
Total score in 2007 DCD questionnaire	48.3 ± 11.5	41.6 ± 12.1	0.250
Coexisting conditions (number and %)			0.898
Attention deficit hyperactivity disorder	10 (21.3%)	10 (24.4%)	
Dyslexia	5 (10.6%)	7 (17.1%)	
Suspected autism-spectrum disorder	12 (25.5%)	13 (31.7%)	
Routine medication for attention deficit hyperactivity disorder (number and %)			0.907
Ritalin	1 (2.1%)	1 (2.4%)	
Concerta	2 (4.3%)	1 (2.4%)	
Unknown	1 (2.1%)	1 (2.4%)	

Note. FMT = Functional Movement Training.

**Table 2:** Changes in outcome variables by group and between-group differences in outcomes at 3 and 6 months.

Outcome	Task-Specific FMT (n = 47)	Control (n = 41)	Between-Group Difference in Change from Baseline (95% CI)			P value		
			Task-Specific FMT Group vs. Control Group	P value	Effect size	Group	Time	Group x Time
Primary outcomes								
SOT somatosensory ratio						<0.001	0.158	0.786
Baseline value	0.96 ± 0.06	0.95 ± 0.07						
Change from baseline								
3 months	0.02 ± 0.04	−0.01 ± 0.02	0.03 (0.02, 0.04)	<0.001	0.95			
6 months	0.02 ± 0.05	−0.01 ± 0.03	0.03 (0.01, 0.05)	<0.001	0.73			
SOT vestibular ratio						0.628	0.467	0.751
Baseline value	0.38 ± 0.16	0.43 ± 0.22						
Change from baseline								
3 months	0.02 ± 0.08	0.01 ± 0.13	0.01 (−0.03, 0.06)	0.569	0.09			
6 months	0.01 ± 0.12	0.00 ± 0.08	0.01 (−0.04, 0.05)	0.742	0.10			
SOT visual ratio						0.663	0.434	0.151
Baseline value	0.61 ± 0.17	0.59 ± 0.22						
Change from baseline								
3 months	0.00 ± 0.07	0.00 ± 0.08	0.00 (−0.03, 0.03)	0.968	0.00			
6 months	−0.01 ± 0.09	0.01 ± 0.08	−0.01 (−0.05, 0.02)	0.412	0.23			
Secondary outcomes								
MABC TIS						0.921	0.853	0.167
Baseline value	15.36 ± 6.52	15.48 ± 4.17						
Change from baseline								
3 months	−0.71 ± 2.91	−0.48 ± 2.19	−0.23 (−1.33, 0.88)	0.684	0.09			
6 months	−0.39 ± 3.08	−0.73 ± 2.59	0.34 (−0.88, 1.55)	0.584	0.12			
MABC balance subscore						0.004	0.680	0.680
Baseline value	2.97 ± 2.07	2.54 ± 1.50						
Change from baseline								
3 months	−1.10 ± 1.54	−0.17 ± 0.64	−0.93 (−1.42, −0.44)	<0.001	0.79			
6 months	−1.00 ± 2.25	−0.17 ± 0.64	−0.83 (−1.52, −0.14)	0.019	0.50			
UST centre of pressure sway velocity (°/s)						0.005	0.130	0.659
Baseline value	2.56 ± 1.30	2.66 ± 2.07						
Change from baseline								
3 months	−0.56 ± 1.21	−0.02 ± 0.45	−0.54 (−0.91, −0.16)	0.006	0.59			
6 months	−0.61 ± 1.17	−0.05 ± 0.31	−0.56 (−0.92, −0.20)	0.003	0.65			

Note. All values are means ± SD unless noted otherwise. The baseline values were comparable between the 2 groups (P > 0.05) according to the results of independent t test. Change scores were calculated as (3-month – baseline) and (6-month – baseline). The overall P values for the outcome measures were derived from two-way repeated measures analysis of variance. P values for between-group comparison of change scores were derived from independent t test, with an overall significance level of 0.05. FMT = Functional Movement Training. CI = confidence interval. SOT = Sensory Organisation Test. MABC = Movement Assessment Battery for Children. TIS = Total impairment score. UST = Unilateral Stance Test.

**Table 3:** 3-month task-specific Functional Movement Training protocol.

Exercise	Details and exercise progression	Frequency	Intensity	Duration
Two-leg balance on foam with electromyographic biofeedback	• Participant stands on a stability trainer. Activity of the rectus femoris and gluteus maximus muscles monitored by electromyographic biofeedback.	Twice per week	Not beyond muscle fatigue	10 minutes
	• Participant learns to maintain balance through coordinated hip and ankle strategies.			
One-leg balance on ground (alternate feet)	• Participant stands on one leg with arms held freely at sides and free leg bent backwards at the knee. Swaying is allowed.		Not beyond muscle fatigue	5 minutes
	• Participant progresses to one-leg balance on balance board with a jumping-stand base (alternate feet).			
Walking in a straight line with heels raised	• Participant walks on tiptoe (heels raised) in a straight line for 4.5 m.		20 repetitions	5 minutes
	• Participant progresses to heel-to-toe (tandem) walking in a straight line (4.5 m).			
Double-leg hops	• Participant jumps forward repeatedly with feet together; each series of jumps must be completed in a balanced, controlled position.		50 hops (per foot)	5 minutes
	• Participant progresses to continuous single-leg hops forward (alternate feet).			
Ball balance while walking	• Participant balances a ball on a peg board while walking. The board must be steadied to ensure that the ball remains stationary without being held. The board can be held in either the right or the left hand.		Walking for 50 metres	5 minutes

Note. Children with DCD practised these balance manoeuvres repeatedly for 1.5 hours in each training session. Short breaks were allowed if absolutely necessary.
