# Supplementary material for: Task-Specific Balance Training Improves the Sensory Organisation of Balance Control in Children with Developmental Coordination Disorder: A Randomised Controlled Trial
Source: Sci Rep. 2016 Feb 11;6:20945. doi: 10.1038/srep20945 (PMC4750073; doi:10.1038/srep20945)
Supplement: Supplementary Information [file srep20945-s1.pdf]

Task-Specific Balance Training Improves the Sensory Organisation of Balance Control in Children with Developmental Coordination Disorder: A Randomised Controlled Trial

Shirley S.M. Fong[1,*], X. Guo[2], Karen P.Y. Liu[3], W.Y. Ki[4], Lobo H.T. Louie[5], Raymond C.K. Chung[2], and Duncan J. Macfarlane[1]

[1]Institute of Human Performance, The University of Hong Kong, Pokfulam, Hong Kong.

[2]Department of Rehabilitation Sciences, The Hong Kong Polytechnic University, Hung Hom, Hong Kong.

[3]School of Science and Health (Occupational Therapy), University of Western Sydney, NSW, Australia.

[4]Health, Physical Education and Recreation Department, Emporia State University, USA.

[5]Department of Physical Education, Hong Kong Baptist University, Kowloon Tong, Hong Kong.

***Correspondence and requests for materials should be addressed to** Shirley S.M. Fong, Institute of Human Performance, The University of Hong Kong, Pokfulam, Hong Kong. Email: smfong@hku.hk; tel.: (852)28315260; fax: (852)28551712.

Short title: Functional-movement training for children with developmental coordination disorder

Supplementary information

	Task-Specific FMT (n=47)	Control (n=41)
**Condition 1 equilibrium score**		
Baseline value	84.52 ± 0.99	81.13 ± 1.50
Value at 3 months	87.00 ± 1.49	85.81 ± 2.20
Value at 6 months	88.33 ± 1.39	88.53 ± 2.01
**Condition 2 equilibrium score**		
Baseline value	81.14 ± 1.13	77.23 ± 1.89
Value at 3 months	85.26 ± 1.55	80.66 ± 3.12
Value at 6 months	86.56 ± 2.33	83.22 ± 2.07
**Condition 3 equilibrium score**		
Baseline value	78.78 ± 1.38	75.06 ± 2.20
Value at 3 months	83.56 ± 1.70	77.86 ± 3.99
Value at 6 months	82.63 ± 1.35	84.13 ± 2.14
**Condition 4 equilibrium score**		
Baseline value	51.56 ± 2.47	47.87 ± 3.91
Value at 3 months	53.07 ± 4.90	50.63 ± 4.50
Value at 6 months	52.99 ± 4.90	53.12 ± 5.24
**Condition 5 equilibrium score**		
Baseline value	31.83 ± 2.29	35.23 ± 3.65
Value at 3 months	34.80 ± 4.40	37.76 ± 6.80
Value at 6 months	34.45 ± 4.56	38.07 ± 7.01
**Condition 6 equilibrium score**		
Baseline value	24.42 ± 3.05	29.95 ± 3.68
Value at 3 months	46.89 ± 4.44	41.05 ± 6.85
Value at 6 months	47.70 ± 5.62	46.60 ± 7.08

Supplementary Table S1. Sensory Organisation Test equilibrium scores of the participants with developmental coordination disorder.

Note. All values are means ± SE unless noted otherwise.
